# Attrition in Interpersonal Psychotherapy Among Women With Post-traumatic Stress Disorder Following Sexual Assault

**DOI:** 10.3389/fpsyg.2019.02120

**Published:** 2019-09-13

**Authors:** Cecília R. Proença, John C. Markowitz, Euthymia A. Prado, Rosaly Braga, Bruno M. Coimbra, Thays F. Mello, Mariana R. Maciel, Mariana Pupo, Juliana Póvoa, Andrea F. Mello, Marcelo F. Mello

**Affiliations:** ^1^Department of Psychiatry, Federal University of São Paulo (UNIFESP), São Paulo, Brazil; ^2^New York State Psychiatric Institute, Columbia University, New York, NY, United States

**Keywords:** interpersonal psychotherapy, sexual assault, post-traumatic stress disorder, adherence, attrition

## Abstract

**Background:**

An estimated 16.9% of adult Brazilian women experience sexual assault in their lifetime. Almost half of women who suffer such trauma develop post-traumatic stress disorder (PTSD). Markowitz et al. (2015) found that an affect-focused non-exposure therapy, Interpersonal Psychotherapy (IPT), adapted to treat PTSD (IPT-PTSD) had similar efficacy to and lower dropout rates than Prolonged Exposure (PE), the “gold standard,” most studied exposure therapy for PTSD.

**Objective:**

To assess attrition rates in IPT of sexually assaulted women recently diagnosed with PTSD.

**Methods:**

The current study derives from a two-arm, randomized controlled clinical trial of sexually assaulted women with PTSD who received 14 weeks of standardized treatment with either IPT-PTSD or sertraline. *Sample*: The 32 patients in the IPT treatment arm were analyzed.

**Results:**

Overall attrition was 29%. One patient was withdrawn because of suicidal risk; four dropped out pre-treatment, and five dropped out during IPT-PTSD. If the excluded patient is considered a dropout, the rate increases to 31%.

**Discussion:**

This is the first formal study of IPT for PTSD specifically due to sexual assault. IPT attrition approximated dropout rates in PE studies, which are often around 30%, and to the sertraline group in our study (34.5%). Further research should compare IPT and PE among sexually assaulted women to clarify our hypothesis that IPT could be an attractive alternative approach for this patient group.

## Introduction

Sexual violence against women is unfortunately widespread. The World Health Organization (WHO) defines sexual violence as any non-consensual sexual activity in which one person uses force or takes advantage of another (World Health Organization [WHO], 2011). It is considered a traumatic event, with high risk for developing post-traumatic stress disorder (PTSD) compared to conditional PTSD risk of other trauma types such as kidnapping (27.5%) or natural disaster (8.4%). Almost half of sexually assaulted women develop PTSD (Foa et al., 1991; Luz et al., 2016), a trauma-triggered psychiatric disorder that entails intense psychological distress and significant social, occupational, and interpersonal dysfunction. Treatment approaches for PTSD include pharmacotherapy and psychotherapy (Chivers-Wilson, 2006), with PTSD guidelines listing trauma-focused psychotherapies such as prolonged exposure (PE) and cognitive therapy using exposure techniques as the first-line treatments (Guideline, 2005; Foa et al., 2008; Forbes et al., 2010; Cusack et al., 2016; Card, 2017). Trauma-focused psychotherapies always include an exposure component: treatment repeatedly exposes the patient to traumatic memories or cues in a safe environment in order to evoke, modify, and habituate to dysfunctional fear responses (Foa and Kozak, 1986). Many traumatized individuals refuse or cannot tolerate this type of treatment, however, as a characteristic of PTSD is to fear and avoid traumatic reminders (Weissman et al., 2007; Markowitz, 2016).

Attrition is a common metric of treatment tolerability (Hill et al., 1992). Dropout rates from PE usually approximate 30%, including among sexually assaulted patients (Foa et al., 1991; Resick et al., 2002; Hembree et al., 2003; Imel et al., 2013; Keefe et al., 2018) ranging across studies from 28 to 68% (Garcia et al., 2011; Gros et al., 2013), possibly due to its aversive nature of exposing patients to traumatic memories or cues. Their high attrition rate may be the most significant barrier affecting the efficacy of trauma-focused therapies (Markowitz, 2016).

Non-exposure psychotherapies should accordingly be considered and tested to treat PTSD. Interpersonal psychotherapy (IPT) is one such option. IPT is a well-known manualized, diagnosis-targeted, and time-limited psychotherapy, with efficacy for depression, eating, and anxiety disorders (Markowitz, 2016). Markowitz et al. (2015) adapted IPT to treat PTSD (IPT-PTSD) and found similar efficacy and lower dropout rates for IPT (15%) than PE (29%), especially among patients diagnosed with comorbid major depression.

Interpersonal psychotherapy has three phases. In the first, the therapist determines the psychiatric diagnosis and the interpersonal context in which the patient lives, exploring current, and past significant relationships. History-taking yields a focus that links the symptoms to the interpersonal context. The therapist offers a formulation of a life crisis on which to focus the treatment (Weissman et al., 2018). Among patients diagnosed with PTSD, the usual treatment foci are role transition, role dispute, or complicated bereavement (Markowitz, 2016). In the middle phase, the therapist uses specific strategies to help the patient resolve the focus, validating the patient’s feelings, encouraging the patient to express his or her thoughts and feelings and to take appropriate social risks, with the goal of improving the ability to assert needs and wishes in relationships (Weissman et al., 2014). In the final sessions, the therapist highlights the patient’s gains during the treatment, reinforcing competence (e.g., as a survivor) rather than the victim role, and helping the patient to experience the end of the psychotherapy process, which has positive and painful aspects (Weissman et al., 2014; Markowitz, 2016).

The IPT adaptation for PTSD emphasizes affective attunement, as patients with PTSD usually report being numb, yet need to read their feelings in order to respond to social interactions and determine whom they can trust. IPT-PTSD focuses not on the trauma itself but its interpersonal after-effects, the often severe social consequences of trauma on the patient’s subsequent life and relationships (Markowitz, 2016).

Despite the deleterious effects that PTSD has on interpersonal relationships and psychosocial functioning, interpersonal features have generally played a marginal role in PTSD treatment approaches (Markowitz and Weissman, 2004). Since PTSD is closely related to stressful interpersonal relationships (Markowitz et al., 2009), focusing on the patients’ relationships seems a reasonable approach to symptom improvement.

The present study is part of a two-arm clinical trial of women who developed PTSD after a sexual assault and received either IPT-PTSD or sertraline for 14 weeks, delivered as standardized treatment, after a baseline interview to evaluate PTSD, depressive and anxiety symptoms. The objective is to assess attrition of non-trauma-focused IPT-PTSD in the subsample of 32 women randomized to IPT-PTSD treatment. We expected IPT would have lower attrition for PTSD than PE, for which it is usually around 30% (Hembree et al., 2003; Imel et al., 2013).

## Materials and Methods

This study was conducted within a larger randomized controlled clinical trial comparing sertraline and IPT-PTSD. The cohort comprises patients diagnosed with PTSD following sexual assault and healthy, age-matched women. The research defined *sexual assault* as rape (forced penetration), attempted rape (attempted forced penetration), or alcohol/drug-facilitated forced penetration or attempted penetration. The term “sexual abuse” in this paper refers only to childhood sexual abuse.

Women enrolled in the study were randomly assigned to receive 14 weeks of treatment with either IPT-PTSD or sertraline, without considering patient preference. The main study aim is to evaluate PTSD-related neuroprogression in the cohort. Recruitment started in 2016 and ended in March 2019.

### Sample

Our sample comprises the 32 women randomized to the IPT-PTSD treatment arm of the clinical trial. Inclusion criteria were (1) women who suffered sexual assault 1–6 months beforehand, regardless of childhood sexual abuse; (2) current PTSD diagnosis according to the Mini International Neuropsychiatric Interview (MINI) (Amorim, 2000) and Diagnostic and Statistical Manual of Mental Disorders, Fifth Edition criteria (American Psychiatric Association, 2013), evaluated using the Clinician-Administered PTSD Scale (CAPS-5; Watanabe et al., 2019), positive if score > 26 (Blake et al., 1995); (3) age 18–45 years old; and (4) signed informed consent approved by the Ethics Review Board. Exclusion criteria were (1) neurologic disease; unstable medical condition; chronic corticosteroid use; substance dependence in remission for less than 6 months; schizophrenia or bipolar disorder; cognitive impairment; (2) ongoing psychiatric or psychotherapeutic treatment; (3) severe suicidal risk, or (4) pregnancy.

### Procedures and Assessments

The primary study referral site was Hospital Pérola Byington (HPB), a specialized women’s health center in São Paulo City. The HPB team referred patients after a short screening for sexual assault and PTSD symptoms using the National Stressful Events Survey PTSD Short Scale (NSESSS) to schedule a screening appointment at Federal University of São Paulo (UNIFESP) with trained researchers. The screening interview included administration of the MINI, the CAPS-5 to confirm PTSD diagnosis and evaluate PTSD severity, the Beck Depression Inventory (BDI), and the Beck Anxiety Inventory (BAI). All study data are stored in REDCap, a secure, web-based application widely used in research for building and managing online surveys and databases.

The 32 eligible women randomized to IPT-PTSD were informed about the psychotherapeutic approach, frequency of sessions, and treatment duration before treatment began.

### Treatment: IPT-PTSD

IPT-PTSD was delivered in 14 weekly, 50-min sessions, following the IPT-PTSD manual (Markowitz, 2016). Instead of focusing on trauma, IPT focuses on interpersonal consequences of the traumatic experience in order to improve social support and interpersonal functioning. Five trained psychotherapists applied IPT-PTSD following the manual (Markowitz, 2016), each therapist accepting patients according to their schedule availability. All IPT therapists were M.D. or Master’s degree psychiatrists, 32–46 years old, who had graduated in psychiatry in the last 2–8 years and were clinically experienced in treating PTSD. All therapists had at least 1 year of experience with IPT to treat depression and were trained and supervised to use IPT to treat patients with PTSD in the trial.

Sessions were audiotaped with patient authorization to certify therapy adherence, and two expert IPT psychotherapists supervised sessions weekly. The supervisors were a Ph.D. psychiatrist and a master’s degree psychologist with more than 10 years of experience in treating patients with IPT and in treating patients with PTSD.

### Statistical Analysis

Treatment adherence was calculated based on the number of patients who completed week 14 of the clinical trial. Women who were randomized to IPT who did not initiate treatment or who started IPT-PTSD but left treatment before week 14 comprised the dropout group.

An initial analysis of variables of interest was carried out, describing absolute (n) and relative (%) frequency distributions for qualitative variables, and the main summary measures, such as mean, standard deviation, median, minimum and maximum values for the quantitative variables.

In order to evaluate patients characteristics to identify possible associations between dropouts and completers, chi-square test was applied to categorical variables (race, marital status, religion, income, childhood sexual abuse, drug facilitated sexual assault, BDI severity, and education level), while the non-parametric Mann–Whitney *U* test was applied to compare continuous variables (age, CAPS, BAI, and BDI) in relation to each continuous variable. The non-parametric bootstrap procedure was used to obtain an interval estimate for the mean, considering 1000 bootstrap samples, inasmuch as the sample size in each group is small. In addition, we reported the effect sizes for Mann–Whitney *U* test (r = z/$\sqrt{n}$) and the Cramer’ V was calculated for each chi-square test.

Significance level was fixed at 5% for all tests. Statistical analyses were performed using R software version 3.5 (R Foundation for Statistical Computing, Vienna, Austria).

## Results

Thirty-two sexually assaulted women were randomized to IPT. One patient was withdrawn after session seven due to severe suicidal ideation. Despite strong attachment with the therapist, and “adherent” treatment (attending all sessions during the 14 IPT weeks), this patient appeared at suicidal risk and hence was excluded from the trial. Receiving pharmacotherapy, she completed the 14 IPT-PTSD sessions combined with medication, meaning IPT was tolerable but ineffective in this case. Four patients dropped out before initiating treatment, and five dropped out during IPT-PTSD. Our team contacted eight out of the nine dropouts to understand why they left treatment. Only one patient, a pre-treatment dropout, refused contact.

The other three patients randomized to IPT-PTSD who did not begin treatment reported that they could not arrange their schedules to accommodate treatment with their daily life and work. One of them added that attending our trauma clinic would remind her of the trauma, so she preferred to seek treatment in a non-specialized center. Among the five women who started IPT-PTSD treatment, two reported they could not tolerate sessions because the stimulus of attending our service, known as a specialized violence center, triggered trauma reminders (these patients left after the third and fifth IPT sessions). The other three patients reportedly dropped out after starting treatment because they could not coordinate treatment with their working schedules, leaving treatment between the fifth and eighth sessions.

**FIGURE 1:**
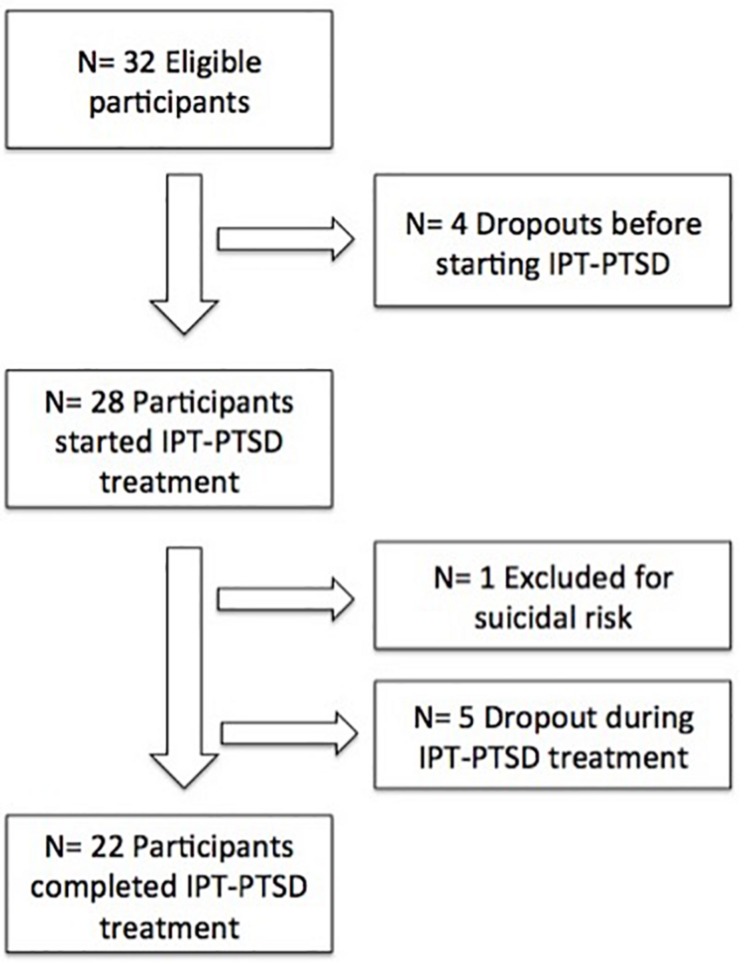
Fluxogram of patients enrolled in the study.

Thus of 32 patients, 22 (69%) completed the 14-week trial of IPT-PTSD. If the withdrawn woman is considered a dropout, the dropout rate increases to 31%. Otherwise, 22 of 31 women completed treatment, a dropout rate of 29% (Figure 1). Patients who initiated IPT-PTSD attended a mean 81% of sessions.

Female therapists treated all patients, excepting one case treated by a male research team member who had to leave the trial after completing this single treatment. Three of five women who dropped out after beginning IPT-PTSD were treated by the same therapist; the other two dropouts each had different therapists. Considering the reasons each patient stated for stopping treatment, we found that the same therapist treated both women who reported they could not tolerate the treatment. These data suggest that some characteristic of this therapist, or of therapist and patient match, might partially explain our attrition result. The other two patients, who left treatment because they started working, were treated by two different therapists.

Of the study’s 32 patients, 30 had a comorbid diagnosis of major depression on the MINI. Two patients had missing MINI depressive symptom data, so we accessed their baseline BDI scores, which were 25 and 38, suggesting our entire sample had comorbid depression at baseline. Depressive comorbidity rates did not differ statistically between the IPT and sertraline groups.

There was no statistically significant difference between dropouts and IPT-PTSD completers in severity of PTSD (*p* = 0.965) or depressive symptoms (*p* = 0.999), drug-facilitated sexual assault (*p* = 0.999), history of childhood sexual abuse (*p* = 0.295), or social demographic characteristics: race, level of education, marital status or religion at the inclusion visit (Supplementary Table S1). When we compared dropouts with women who completed treatment, the correlation between dropouts and family income reached trend level statistical significance (*p* = 0.095) (Supplementary Table S1). Age of study patients ranged from 19 to 42, with no statistically significant difference between dropouts and IPT-PTSD completers (*p* = 0.443) (Supplementary Table S1), although small numbers limit the power to detect such differences. There was no statistically significant difference between IPT arm dropout rate (29%) and the sertraline arm dropout rate (34.5%) (*p* = 0.45).

## Discussion

Psychotherapy attrition among patients diagnosed with PTSD is a major clinical challenge (Bradley et al., 2005; Perkonigg et al., 2005). A significant proportion of patients drop out prematurely, a concern because untreated PTSD is associated with poor prognosis (Keefe et al., 2018). Few studies have tried to identify specific characteristics of patients that could predispose to completing or not completing a particular treatment.

Markowitz et al. (2017) published the first RCT using individual IPT for PTSD in 2015. This trial included patients who suffered different trauma types, including a subset (35%) with “sexual trauma” (as defined by the CAPS Life Events Checklist). Patients diagnosed with PTSD and comorbid major depression were more likely to complete IPT treatment than PE (Markowitz, 2016). Markowitz et al. (2017) analyzed the subsample of sexually traumatized patients in that RCT and found that IPT had better outcomes than PE or Relaxation Therapy. No difference emerged for other trauma types. The authors reported that sexual trauma moderated the effect of treatment on PTSD clusters B (reexperiencing) and D (hyperarousal) symptoms, with greater improvement in the IPT group.

Considering these previous findings, we designed a study including only sexually assaulted women and evaluated IPT-PTSD attrition. This is the first formal study in the literature using IPT-PTSD only for sexually assaulted women. We expected IPT would benefit this patient group with lower attrition than historic exposure therapy rates. However, we found dropout rates similar to research applying PE for sexually assaulted women (Foa et al., 1991; Nishith et al., 2002; Resick et al., 2002; Keefe et al., 2018). To understand these findings, we evaluated variables measured in our research related to sample characteristics, therapist profiles, and psychopathology factors. Small sample size may have limited our likelihood of finding statistically significant results.

One of our findings is that the performance of one therapist could partially explain the high dropout rate among women who started IPT: she treated 60% of women who started and dropped out of IPT. This therapist had only one of four patients complete treatment (25%), whereas other therapists had completion rates as high as 90 and 100%; indeed, everyone else had at least a 75% completion rate. We could not find any explanation for this higher dropout rates. It is clinically recognized that different therapists, despite similar training, have different capabilities with different kinds of patients.

The qualitative data we collected from patients who did not complete IPT-PTSD allows us to theorize that another possibly important factor related to the high attrition rate were difficulties in accommodating treatment to daily life activities and work (reported by 67% women who left treatment) and PTSD symptomatology itself (reported by 33% of patients). We should recall the context in which this research took place. With a population over 20 million, São Paulo is one of the world’s largest metropolises, with very limited and commonly overcrowded public transportation. Most women in the trial relied on public transportation to reach our service, spending 1–2 h en route from their houses. Long distances and crowded buses and trains might impede PTSD patients, especially considering that our sample of crowd-averse women had been very recently sexually assaulted.

Some difficulties in completing IPT treatment seem related to the psychopathology of PTSD itself. For some patients, the trauma clinic treatment setting was itself sufficient stimulus to evoke the trauma, precluding tolerating even a non-trauma focused psychotherapy. In their New York civilian subsample of sexual trauma patients (*N* = 17), Markowitz et al. (2015) found only a 6% attrition rate in IPT (J. Markowitz, personal communication, May 2019). Despite the small sample, the disparity between these findings might reinforce our hypothesis that the effect of one therapist could also have affected our observed attrition rates, in addition to transportation and economic factors that might have impeded any kind of regular treatment in our social context.

This trial has limitations, including small sample size and the absence of an exposure therapy arm to allow direct comparison of attrition rates for different psychotherapy approaches in the same group of women. More research is necessary to understand this specific population and its retention in treatment, perhaps using a mixed methods approach. Although we cannot state that IPT-PTSD has lower dropout rates than exposure therapies for this specific population, it appears no worse; IPT-PTSD could be considered an alternative non-trauma-focused psychotherapeutic approach.

In light of little research exploring benefits of non-trauma-focused PTSD treatments, and recent findings demonstrating that trauma type may moderate psychotherapy outcome in PTSD (Markowitz et al., 2017), our results suggest that the dropout rate is no worse than it historically is for PE. We believe it crucial to spread the knowledge of an attractive alternative psychotherapeutic intervention for this particular group of traumatized women. Although our findings do not suggest that IPT is immune to the same challenges as PE and other exposure therapies, further research should evaluate psychotherapy approaches, in the same socioeconomic context, particularly with this group of patients.

## Data Availability

The datasets generated for this study are available on request to the corresponding author.

## Ethics Statement

The studies involving human participants were reviewed and approved by the Comite de Ética em Pesquisa (CEP) – UNIFESP. The patients/participants provided their written informed consent to participate in this study.

## Author Contributions

CP, JM, MFM, and AM worked on analyses, design, and writing. EP, RB, BC, TM, MRM, MP, and JP contributed in collecting the data and proofreading. AM and MFM shared the last position in the present manuscript. All authors listed have contributed to the intellectual development and writing of this article, and read and approved the final version of this article.

## Conflict of Interest Statement

The authors declare that the research was conducted in the absence of any commercial or financial relationships that could be construed as a potential conflict of interest.
