# Supplementary material for: Attrition in Interpersonal Psychotherapy Among Women With Post-traumatic Stress Disorder Following Sexual Assault
Source: Front Psychol. 2019 Sep 13;10:2120. doi: 10.3389/fpsyg.2019.02120 (PMC6753915; doi:10.3389/fpsyg.2019.02120)
Supplement: Supplementary file 1 [file Table_1.DOCX]

**Table 1: Baseline qualitative and quantitative characteristics of study sample**

| **Variable** | **Category** | **IPT Completed** | | **Total** | **Statistic^#^** | **Degree of freedom or Z** | **p** | **Effect size*** |
| --- | --- | --- | --- | --- | --- | --- | --- | --- |
|  |  | **No** | **Yes** |  |  |  |  |  |
| **Race** | White | 6 (66.7%) | 6 (27.3%) | 12 (38.7%) | 4.178 | 1 | 0.056 | 0.367 |
|  | Other | 3 (33.3%) | 16 (72.7%) | 19 (61.3%) |  |  |  |  |
| **Marital Status** | Single | 8 (88.9%) | 16 (72.7%) | 24 (77.4%) | 1.474 | 2 | 0.674 | 0.218 |
|  | Married | 1 (11.1%) | 3 (13.6%) | 4 (12.9%) |  |  |  |  |
|  | Stable Union | 0 (0%) | 3 (13.6%) | 3 (9.7%) |  |  |  |  |
| **Religion** | Catholic | 2 (22.2%) | 6 (28.6%) | 8 (26.7%) | 2.880 | 4 | 0.677 | 0.310 |
|  | Evangelic | 2 (22.2%) | 8 (38.1%) | 10 (33.3%) |  |  |  |  |
|  | Spiritist | 2 (22.2%) | 1 (4.8%) | 3 (10%) |  |  |  |  |
|  | No religion | 2 (22.2%) | 5 (23.8%) | 7 (23.3%) |  |  |  |  |
|  | Other | 1 (11.1%) | 2 (9.6%) | 3 (6.7%) |  |  |  |  |
| **Income (IBGE 2016)** | R$ 1874,00 or less | 6 (66.7%) | 20 (90.9%) | 26 (83.9%) | 5.363 | 2 | 0.095 | 0.416 |
|  | R$ 1874,01-3784,00 | 1 (11.1%) | 2 (9.1%) | 3 (9.7%) |  |  |  |  |
|  | R$ 3748,01 or higher | 2 (22.2%) | 0 (0%) | 2 (6.5%) |  |  |  |  |
| **Childhood sexual abuse** | No | 9 (100%) | 18 (81.8%) | 27 (87.1%) | 1.879 | 1 | 0.295 | 0.246 |
|  | Yes | 0 (0%) | 4 (18.2%) | 4 (12.9%) |  |  |  |  |
| **Drug facilitated sexual assault** | No | 6 (66.7%) | 15 (68.2%) | 21 (67.7%) | 0.007 | 1 | 0.999 | 0.015 |
|  | Yes | 3 (33.3%) | 7 (31.8%) | 10 (32.3%) |  |  |  |  |
| **BDI** | Minimun/Mild | 3 (33.3%) | 7 (31.8%) | 10 (32.3%) | 0.007 | 1 | 0.999 | 0.015 |
|  | Moderate/Severe | 6 (66.7%) | 15 (68.2%) | 21 (67.7%) |  |  |  |  |
| **Education Level** | More than 12 years | 5 (55.6%) | 11 (50%) | 16 (51.6%) | 0.079 | 1 | 0.999 | 0.050 |
|  | Less than 12 years | 4 (44.4%) | 11 (50%) | 15 (48.4%) |  |  |  |  |
| **Age** | Mean (SD) | 22.44 (3.88) | 25.32 (7.53) | 24.48 (6.74) | 81.5⁺ | -0.767 | 0.443 | -0.138** |
|  | Median (Min-Max) | 23 (19-31) | 22 (19-42) | 22 (19-42) |  |  |  |  |
|  | 95% CI*** | 20.14 to 25.37 | 22.41 to 28.92 | 22.2 to 27.10 |  |  |  |  |
| **CAPS V0** | Mean (SD) | 42.78 (10.21) | 42.36 (9.03) | 42.48 (9.22) | 98⁺ | -0.044 | 0.965 | -0.008** |
|  | Median (Min-Max) | 43 (28-59) | 43 (28-56) | 43 (28-59) |  |  |  |  |
|  | 95% CI*** | 36.50 to 49.55 | 38.36 to 46.50 | 39.16 to 45.90 |  |  |  |  |
| **BAI V0** | Mean (SD) | 25.44 (12.19) | 30.55 (13.01) | 29.06 (12.79) | 76⁺ | -1.002 | 0.316 | -0.18** |
|  | Median (Min-Max) | 24 (9-42) | 34.5 (4-50) | 29 (4-50) |  |  |  |  |
|  | 95% CI*** | 17.11 to 33.54 | 25 to 36.16 | 24.42 to 33.74 |  |  |  |  |
| **BDI V0** | Mean (SD) | 25.22 (12.70) | 29.64 (12.90) | 28.35 (12.79) | 81⁺ | -0.785 | 0.432 | -0.141** |
|  | Median (Min-Max) | 25 (9-47) | 28.5 (10-48) | 27 (6-48) |  |  |  |  |
|  | 95% CI*** | 17.29 to 33.99 | 24.20 to 35.00 | 23.78 to 32.84 |  |  |  |  |

*Cramer’V, **r= z/$\sqrt{n}$ ,*** 95% CI Bootstrap obtained on 1000 bootstrap samples, **^#^** Chi-square statistic and ⁺Mann-Whitney U statistic.
